# Textile-Friendly Interconnection between Wearable Measurement Instrumentation and Sensorized Garments—Initial Performance Evaluation for Electrocardiogram Recordings

**DOI:** 10.3390/s19204426

**Published:** 2019-10-12

**Authors:** Fernando Seoane, Azadeh Soroudi, Ke Lu, David Nilsson, Marie Nilsson, Farhad Abtahi, Mikael Skrifvars

**Affiliations:** 1 Swedish School of Textiles, University of Borås, 501 90 Borås, Sweden; azadeh.soroudi@hb.se (A.S.); mikael.skrifvars@hb.se (M.S.); 2 Department of Medical Care Technology, Karolinska University hospital, 14 157 Huddinge, Sweden; 3 Institute for Clinical Science, Intervention and Technology, Karolinska Institutet, 141 57 Huddinge, Sweden; farhad.abtahi@ki.se; 4 Department of Electrical Engineering, Chalmers University of Technology, 412 96 Göteborg, Sweden; 5 RISE Research Institutes of Sweden, Box 857, 501 15 Borås, Sweden; david.nilsson@ri.se (D.N.) marie.nilsson@ri.se (M.N.); 6 School of Engineering Sciences in Chemistry, Biotechnology and Health, KTH Royal Institute of Technology, 141 57 Huddinge, Sweden

**Keywords:** conductive polymers, wearable technology, smart textiles, textile–electronic integration

## Abstract

The interconnection between hard electronics and soft textiles remains a noteworthy challenge in regard to the mass production of textile–electronic integrated products such as sensorized garments. The current solutions for this challenge usually have problems with size, flexibility, cost, or complexity of assembly. In this paper, we present a solution with a stretchable and conductive carbon nanotube (CNT)-based paste for screen printing on a textile substrate to produce interconnectors between electronic instrumentation and a sensorized garment. The prototype connectors were evaluated via electrocardiogram (ECG) recordings using a sensorized textile with integrated textile electrodes. The ECG recordings obtained using the connectors were evaluated for signal quality and heart rate detection performance in comparison to ECG recordings obtained with standard pre-gelled Ag/AgCl electrodes and direct cable connection to the ECG amplifier. The results suggest that the ECG recordings obtained with the CNT paste connector are of equivalent quality to those recorded using a silver paste connector or a direct cable and are suitable for the purpose of heart rate detection.

## 1. Introduction

Wearable garments for the measurement of vital signs and biosignals are believed to be a primary part of more personalized, pervasive healthcare to tackle the problems arising with the aging population. During the last decade, several wearable systems including textile electrodes have been reported [1,2,3] and are even being used in commercial products, e.g., Hexoskin T-shirts and Equivital vests. However, there still are issues with the textile–electronic integration, limiting the mass production of such sensorized garments and delaying the market growth forecasted for wearable technology. Manufacturing solutions for interconnecting sensors and wearable instrumentation, such as intarsia knitting with conductive yarn [4], have been proposed. However, these solutions are inadequate for the mass production of embedded cables in the garments, and there are still other key areas where practical solutions for textile–electronic integration are required.

**Figure 1:**
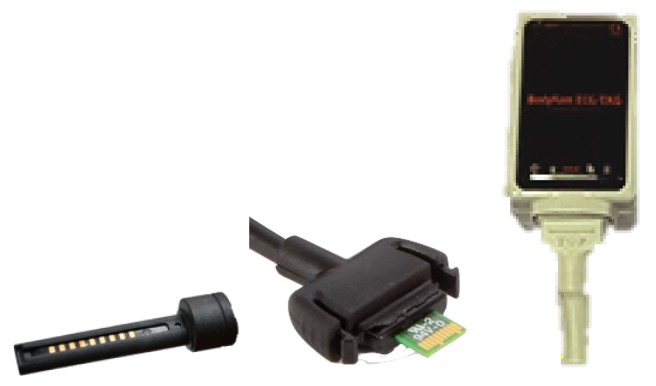
Lead interconnections in wearable instrumentation made in hard plastic from three different recorders: Hexoskin, Equivital, and Bodykom from left to right.

Interconnections between leads and instrumentation are required to be both electrically and mechanically robust. In addition to such common requirements, when connecting electronics in sensorized garments, the interconnection must stand mechanical pulls resulting from the weight of the device and the natural wearing of the garment itself. Moreover, given the chances for rain or an excess of sweat during wear, another requirement would be moisture resistance or even waterproofing. Interconnectors fulfilling these requirements are often found in the field of electronics; however, unfortunately, they are usually costly, bulky, and not stretchable (see Figure 1). 

Most importantly, they are completely foreign to the textile manufacturing process, adding more hurdles to the already challenging process of textile–electronic integration [5].

Screen printing in textile manufacturing is a widespread and established process worldwide [6]; conductive pastes are being continuously developed due to the printed electronics phenomena boosted in the last decade [7]. While most conductive pastes and inks are based on expensive silver or toxic copper [8], both with no elastic properties, carbon-based materials provide electrical conductivity and elastic possibilities [9,10,11,12]. Therefore, combining the elastic and conductive properties of carbon nanotubes (CNTs) [13,14] with screen printing technology seems feasible and has the required potential for enabling textile–electronic integration.

Wearable systems including electrocardiogram recordings have high potential for long-term electrocardiogram recordings. Such recordings have high potential for the screening, diagnosis, and monitoring of different medical conditions, e.g., atrial fibrillation [15,16], autonomic nervous system imbalance, risk assessment of physical workload [17], and sport applications, e.g., estimating energy expenditure [18,19].

In this paper, we introduce the concept of being “textile-friendly” to refer to those methods and techniques for integrating electronics with textiles that take into the account the intrinsic features of textiles—soft, drapable, etc.—and utilize common methods currently accepted in textile manufacturing or implement techniques especially well-suited for soft, light, and drapable materials. As an example of textile-friendliness, the authors present a solution for interconnecting soft textile conductive elements and hard electronic instrumentation components with a fabric printed with an elastic and conductive CNT-based paste. Electrocardiogram (ECG) recordings obtained with the prototype interconnector were studied with the aim to assess the functional performance of the textile–electronic interconnection.

## 2. Materials and Methods 

The experimental measurements were taken after informed consent was given, according to ethical approval no. 274-11 granted by the Ethical Review Board of Gothenburg, Sweden.

### 2.1. Polymer-Based Conductive Elastic Paste

The conductive paste was developed at the University of Borås, and it is based on a stretchable polymer compound formulated with multi-walled carbon nanotubes (MWCNTs) (Chengdu organic chemicals, P.R. China) as the conductive component, proper additives to promote homogienity/printability, and polyurethane (PU) as the binder. The paste manufacturing process was optimized regarding material compositions and mixing parameters (speed and duration), targeting conductivity, homogeneity, and printability parameters including viscosity, dispersion quality, and nominal sheet resistivity in a relaxed state and after elongation. The process started with 10 min high-speed mixing using an overhead mixer equipped with a bladed propeller stirrer, IKA RW 20 Digital Dual-Range Mixers, Germany. After mixing and homogenizing using magnetic stirring at room temperature for 13 h, the prepared mix of the PU, CNTs with water, and additives was filtered through an 80 μm mesh, and the printable MWCNT paste was obtained.

The surface impedance of the developed paste was characterized for different values of thickness and elongation, applying a four-probe method using a Keithley 2000 digital multimeter (Keithley Instruments, Ohio, USA).

### 2.2. Connectors

**Figure 2:**
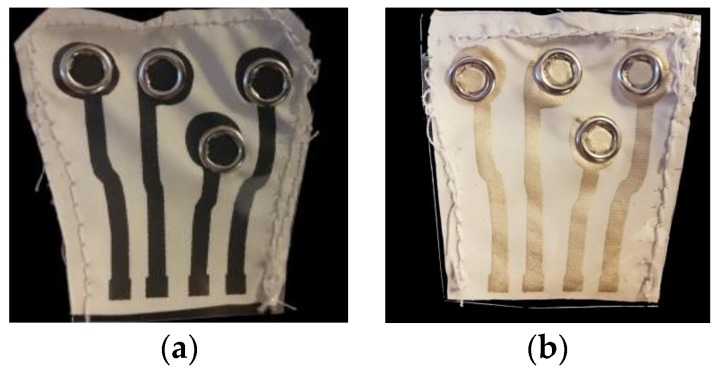

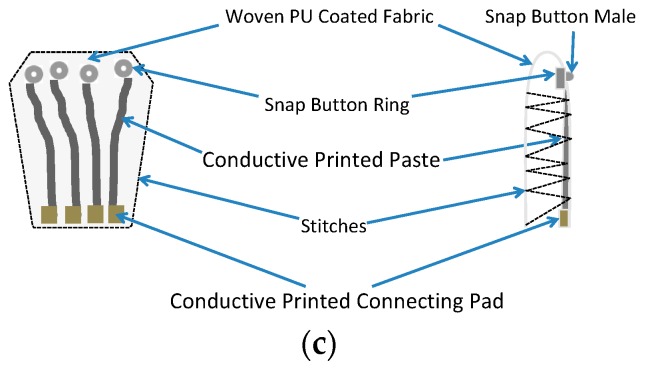
(**a**) The connector printed with multi-walled carbon nanotube (MWCNT)-based paste; (**b**) the connector printed with silver paste; and (**c**) a schematic representation of a printed connector on the fabric with all its textile elements.

The connector was prototyped at the University of Borås using screen-printed fabrics and snap buttons as shown in Figure 2a–c. The fabrics were printed with the conductive pastes in a semiautomatic screen printer from DEK at Acreo, Printed Electronics Laboratory, Norrköping, Sweden. The woven fabric 4191F (F.O.V. Fabrics AB, Sweden) was coated with PU, and it was stretchable in one direction. The conductive pastes used were the developed MWCNTs and the silver-based Dupont Conductor 5000 (DuPont, Delaware, USA).

The conductive paste was printed over the PU-coated side of the 4191F fabric and male snap buttons were pinned through the circular printed pads as shown in Figure 2a,b. The snap buttons were intended for garment interconnection, while the square pads on the edge of the fabric were intended for connecting the measurement lead points of the wearable measurement instrumentation.

The screen-printed fabrics were cured in a fume hood for 120 min at room temperature for the MWCNT paste and 16 min at 100 °C for the Conductor 5000.

The sister connector using the Dupont 5000 paste was produced to evaluate the influence of the intrinsic electrical conductivity on the functional performance of the connector.

The junction impedance was measured using a Keithley 2000 digital multimeter (Keithley Instruments, Ohio, USA) using the two-probe method over three sets of textile–electronic interconnectors in static conditions and during elongation and folding.

### 2.3. Sensorized Garment

**Figure 3:**
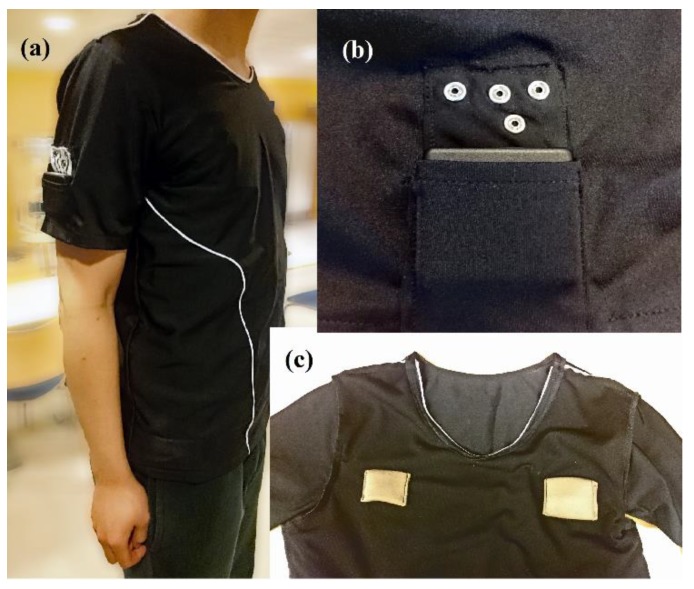
The sensorized garment with textile–electronic interconnector is worn by the test subject in (**a**). (**b**) Snap buttons on the sleeve for the connector were placed above the pocket for a wearable instrument. (**c**) Textile electrodes were sewn to the T-shirt interior, sensorizing the garment.

The connectors were tested with a sensorized T-shirt with sewn textile electrodes in the inner layer of the garment, as shown in Figure 3c. With the sole purpose of ensuring a high-quality ECG measurement and avoiding any potential source of variability coming from the electrodes or the textile interconnection, commonly and extensively used solutions were selected in the construction of the sensorized garment.

The electrodes were made with conductive Shieldex® P130+B fabric manufactured by STATEX Gmbh (Bremen, Germany) of size 6 × 4 cm [20]. The position of the electrodes allows for performing a lead I ECG recording, i.e., a *lateral bipolar recording*, which is also called “right arm to left arm”. The electrodes were connected through copper cables to the female snap buttons on the sleeve as shown in Figure 3b for interconnection with the screen-printed connectors. Note that the only elements in contact with the skin are the textile electrodes, see Figure 3c, which were made with a silverized cotton fabric certificated as biocompatible by the manufacturer; therefore, no additional biocompatibility testing was required.

### 2.4. Experimental Setup

The evaluation was done by comparing the ECG recordings obtained with the MWCNT-based and the silver-based connectors with ECG recordings obtained with Ag/AgCl repositionable red-dot^TM^ 2670-5 gel electrodes manufactured by 3M of size 4 × 3.2 cm. The recordings were obtained with an ADAS1000 evaluation board (Analog Device, Inc., Massachusetts, United States) configured for simultaneous recordings of two independent continuous measurement channels at sampling frequency of 2 kHz. The data collection was done by a Windows application, and the ECG recordings were saved and imported to MATLAB 2017 (The Mathworks, Inc., Massachusetts, United States) for analysis and comparison.

To allow a full and accurate comparison of the obtained ECG recordings using the MWCNT connector, beyond signal morphology in time and spectral content, the gel electrodes were placed as closely as possible to the textile electrodes to record simultaneous ECG measurements. These measurements were used to perform maximum value on the R-wave in each ECG complex (R-peak) detection, which is critical to any heart rate and heart rate variability application. The experimental recordings were done in two steps:

1. Simultaneous synchronized ECG recording with gel electrodes and the sensorized garment using the MWCNT connector;
2. Simultaneous synchronized ECG recording with gel electrodes and the sensorized garment using the Ag connector.

The recordings were performed 10 times for a minimum of 20 s each. The subject remained seated during the experiment with a normal breathing pace at rest. 

### 2.5. ECG Processing

The measurements were preprocessed for powerline interference, baseline wander, and white noise removal. Removed components in each step were analyzed for comparison. The powerline interference was removed by zero-phase forward–backward filtering with an infinite impulse response notch filter (notch@50 Hz, 2 Hz bandwidth). The baseline wander was estimated by the lowest coarse approximation of the discrete wavelet transform. The symlet wavelet and a decomposition level of 10 were used according to [21]. The remaining noise including white noise was removed via the wavelet denoising method. With the purpose of comparing the morphological patterns of recordings, the SURE [22] thresholding rule and hard thresholding method were used to minimize the denoising error in waveform complex formed by the Q, R and S waves on a ECG complex (QRS) complex analysis [23]. R-peaks were detected on the preprocessed recordings using a Pan-Tompkins QRS detector [24].

### 2.6. Material Characterization

The morphology of the printed conductive paste surfaces was characterized by scanning electron microscopy using a Hitachi s-4800 FE-SEM. The acceleration voltage was 5 kV, and the surfaces were coated with chromium before microscopy.

### 2.7. Signal Comparison

Powerline interference and the baseline wander level and R-peak amplitude were compared among each setup. The powerline interference level was measured by calculating the Root Mean Square (RMS) value of the removed 50 Hz component. The difference between the maximum and minimum values on baseline in a 10-second window was used to indicate the baseline wander level for a short period.

A visual ECG morphology comparison was implemented on ensemble-averaged ECG complex data, including waverform complex formed all waves present on a ECG complex except U (PQRST) waveforms, representing the heart’s electrical activity [25]. All PQRST complexes in a 15-second segment were extracted, summed with the alignment of their R-peak, and then averaged [26]. The averaged signals, with non-repetitive interference removed, can indicate systematic morphological differences.

The similarities between recordings from the textile electrodes with MWCNT/Ag connectors and the corresponding synchronized gel recordings from the gel electrode were examined in both time and frequency domains. The time series linear correlation was measured using Person’s correlation coefficient defined by the covariance of the two series over the product of their standard deviations. Cross-spectrum coherence was used for frequency domain comparison. The spectrum was acquired using Welch’s overlapped averaged periodogram and the magnitude-squared coherence was calculated.

The agreement of the Time between R peak in consecutive ECG complexes (RR Intervals) series extracted from the recordings obtained with the textile electrodes using MWCNT-based and silver connectors and the corresponding synchronized recordings obtained with the gel electrodes was evaluated by calculating the Lin’s concordance correlation coefficient [27].

## 3. Results

### Electrical Characterization of the MWCNT Elastic Paste

**Figure 4:**
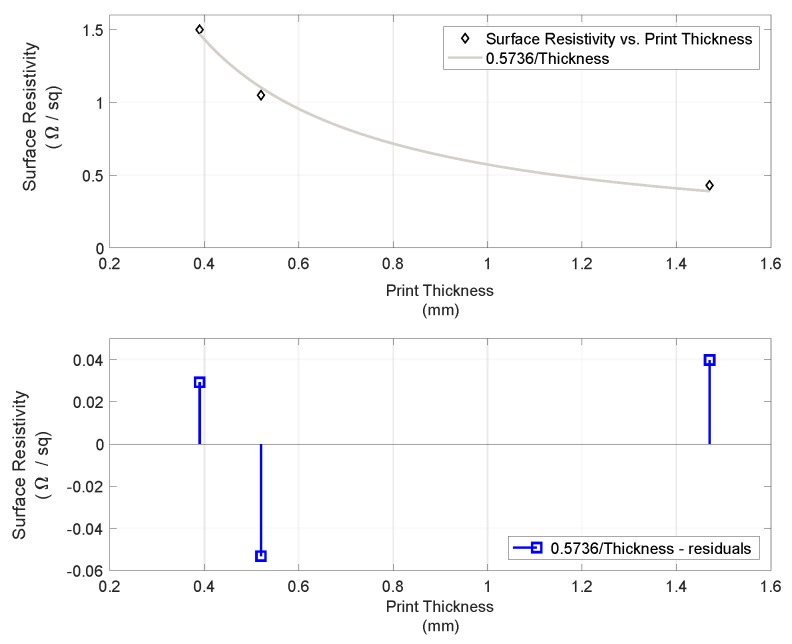
Surface resistivity vs. thickness of lines printed with MWCNT paste in the upper panel and residuals from the fitted function Surface Resistivity = 0.573601/Thickness.

The obtained MWCNT paste had material composition Water/PU/CNT distributed as 1/5.64/1.38 and exhibited a surface resistivity of 350 Ω/sq when spread on a PU-coated polyester textile substrate. In screen-printed lines on the same substrate, the paste showed a surface resistivity that decreased with increasing thickness of the print, as shown in Figure 4. The relation between the resistance values in Ω/sq and the thickness of the print exhibited the expected power relationship denoted by R_s_ = 0.573601/FilmThickness (mm) with a coefficient of determination (R-square) of 0.991. The measured values showed a volume resistivity of 0.37 Ω/mm.

**Figure 5:**
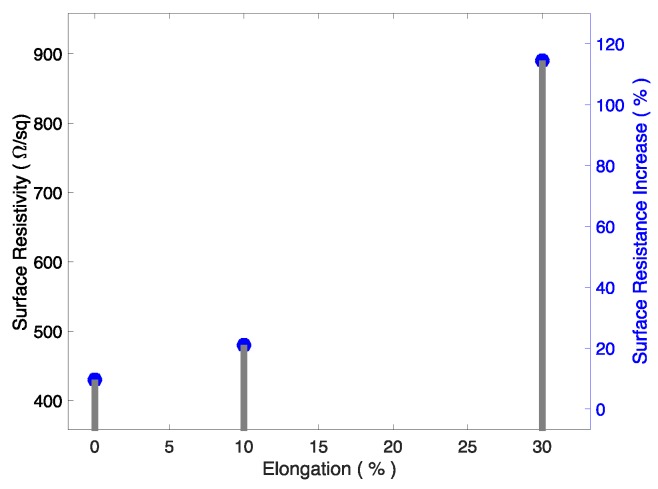
Surface resistivity under elongation.

In Figure 5, we can observe that the surface resistivity increased in a nonlinear manner with elongation. For small elongations, the exhibited increase was similar to the amount of stretch (11% resistance increase for 10% stretch); however, for larger elongation, the resistance was proportionally larger (95% for a 30% stretch).

**Figure 6:**
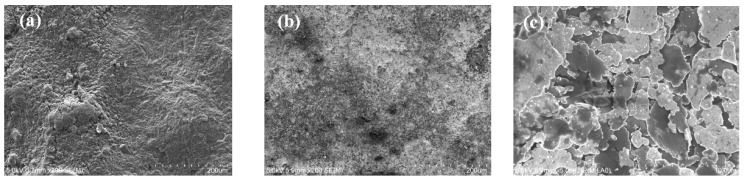
Printed conductive paste surface coatings: (**a**) the MWCNT-based coating; (**b**,**c**) the Conductor 5000 based coating. a and b are with 200 times magnification while c is shown with 10K magnification.

The microscopic images of the material, see Figure 6a–c, show that the connector printed with the MWCNT-based paste had an uneven surface with agglomerates, while the connector printed with the Conductor 5000 had a much more even surface character. At larger magnification, it can be seen that this paste had a flake-like structure with smaller particles.

The electrical resistance of the connector measured before, during, and after folding is reported in Appendix A. As shown in Table 1, it seems that there was a slight increase of resistivity in the mean resistance of the connector with the number of folds.

The increment is clear in the impedance of the connector made with silver paste with an already visible increase from the first 10 folds.

A typical sample of a 5-second recording is shown in Figure 6, where ECG recordings from gel electrodes and textile electrodes with the MWCNT connector are shown together (synchronized).

**Figure 7:**
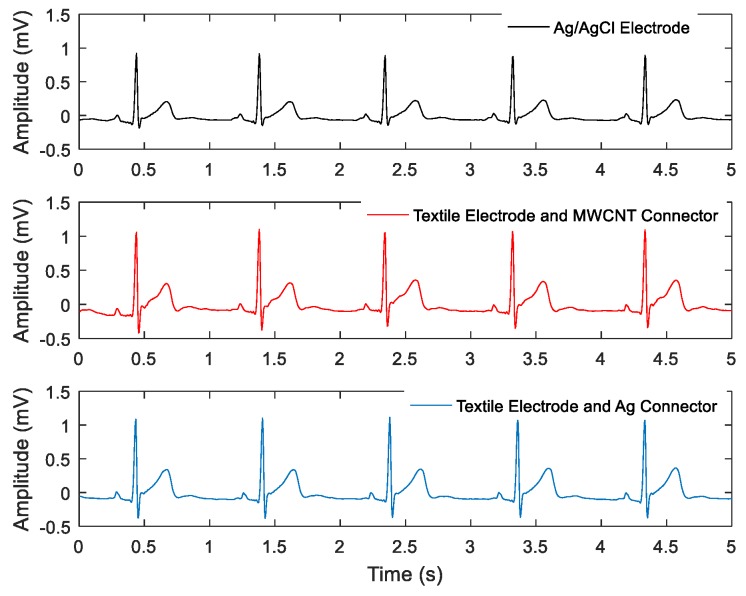
Five-second preprocessed ECG recordings obtained with the three setups.

Figure 7 shows the preprocessed recordings in different setups, where the powerline interference and baseline wander were completely removed in all cases. A selection of calculated parameters is displayed in Table 2. The high correlation coefficients indicate that the recordings from textile electrodes with MWCNT and Ag connectors have a near-perfect linear similarity with the gel electrode recordings. The textile electrode and MWCNT connector set had the most power line interference, followed by the textile electrode and silver connector. The textile electrodes had a significantly larger baseline wander caused by the relative shifting between electrodes and skin caused by breathing movement [28]. The textile electrode gave higher peak amplitude than the gel electrodes, which may be caused by slight differences in position (2−3 cm) and the doubled contacting area for the textile electrodes.

**Figure 8:**
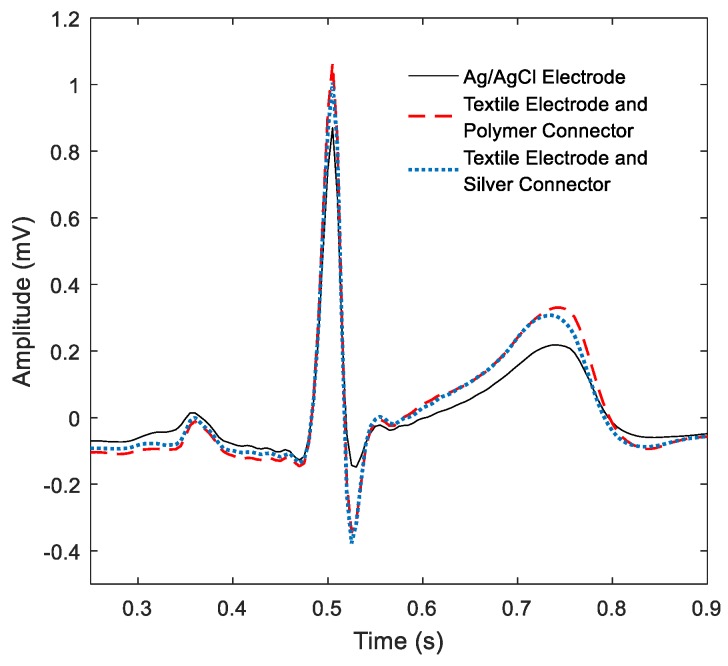
Comparison of the averaged PQRST waves from a 15 s segment.

**Figure 9:**
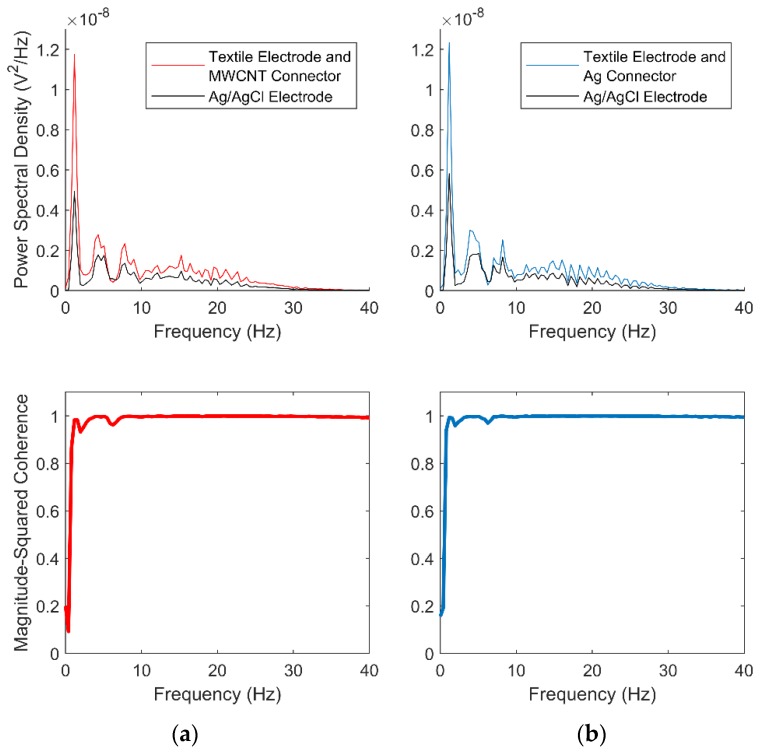
Spectrum content (upper panel) and cross-spectrum coherence (bottom panel) between ECG recordings obtained with gel and textile electrodes with the MWCNT connector vs. gel electrodes (**a**) and with gel and textile electrodes with the Ag connector vs. gel electrodes (**b**).

**Figure 10:**
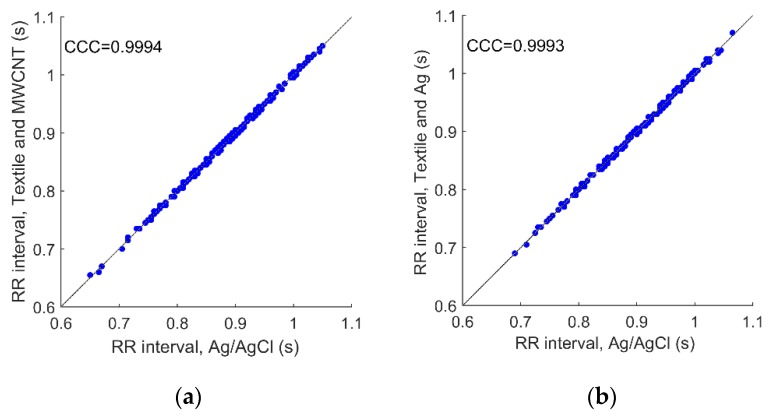
Regression plots of RR Intervals calculated from simultaneous ECG recordings: (**a**) gel electrodes with direct cable connection versus textile electrode using the MWCNT connector; (**b**) gel electrode with direct cable connection versus textile electrode using the silver connector.

The average resistance values of the textile–electronic connectors were 7.47 Ω and 10.47 kΩ for the Ag and the MWCNTs, respectively. Averaged PQRST waves are shown in Figure 8. The waveforms from the MWCNT and Ag connectors are almost identical. The textile electrode recordings had slightly higher amplitude in all waveforms and sharper peaks for R and S waves when compared to the Ag/AgCl electrode recordings. We can observe the amplitude of the peaks in the obtained recordings plotted in Figure 9 and their spectral content in Figure 10. These indicate that the skin–electrode interface in the case of the Ag/AgCl electrodes produced a low-pass filter with a cut-off frequency lower than in the case of textile electrodes, resulting in a smoother PQRST complex.

Figure 9 shows the results of the frequency domain analysis. The cross-spectrum coherence plots exhibit two similar spectra from 1 to 40 Hz in both cases, with a slight difference in the amplitude of the spectral components at all frequencies.

The comparison of extracted RR Intervals is shown in Figure 10. The results from the textile electrodes with MWCNT and Ag connectors have equally good agreement with the gel electrode recordings, with Lin’s concordance correlation coefficients of 0.9994 and 0.9993, respectively.

## 4. Discussion

The prepared MWCNT/polyurethane paste showed a good-quality print on the PU-coated fabric used as a substrate for the connector. The MWCNT material in the interconnector makes the interconnector potentially toxic if it contacts the skin. In such a case, if used as an electrode material, for instance, biocompatibility testing would be been necessary to assess the actual toxicity levels and whether recommendations against their use should be made. Fortunately, in the application presented in this work, it is an interconnector placed outside the sensorized garment, actually connected to the recording device; therefore, between that and the fact that the folding of the PU-coated fabric actually packages the conductive material between two layers of PU, toxic material leaving the interconnector is practically impossible.

### 4.1. Resulting Electrical Properties of the Connectors

The obtained level of sheet resistivity was quite good when compared with the values previously reported by other authors. Super-aligned CNT films showed a resistance of 1 kΩ/sq before stretch and 1.6 kΩ/sq after 60% stretch [29]. poly(3,4-ethylenedioxythiophene) (PEDOT): poly(styrenesulfonate) (PSS) sandwiched between poly(3-hexylthiophene) (P3HT): phenyl-C61-butyric acid methyl ester (PCBM) and Polydimethylsiloxane (PDMS) (buckled) presented a resistivity of 750 kΩ/sq, which was stretchable up to 25% [30]. However, long Ag nanowires on Ecoflex showed resistivity of 9−70 kΩ/sq in prepared stretchable electrodes of a very long Ag nanowire percolation network made by Lee et al. [31].

The observed nonlinear increase in the surface resistivity with elongation was reported by Xu and Zhu in [32] and by Suikkola et al. in [33]; the ability of the MWCNT paste to increase only 11% in surface resistivity for a stretch of 10% is very attractive, especially for this kind of interconnecting application, since it is not expected that a connector needs to stretch far beyond 10%−20%.

The reported increase in the resistance of the connectors suggests that there is certain influence of bending on the printed tracks. The obtained values fall within the range of values reported recently by Du et al. in [34] for bended tracks screen printed with nanowire-based screen printing paste and by Maheshwari et al. in [35] for Ag-based paste. Unfortunately, the sample size does not allow for performing any statistical significance analysis, and in order to produce any accurate further statements on eventual crack formation, SEM images of the connectors after the folding has been performed must be obtained.

### 4.2. Performance as an Interconnector

Despite the differences in volume resistivity of conductive compounds (20.681 Ω/sq/mil for the MWCNT paste and 15 mΩ/sq/mil for the Dupont Conductor 5000 [36]) and in the total resistance of the interconnector (10.47 kΩ and 7.47 Ω for the MWCNT paste and the Dupont Conductor, respectively), the results have shown that there is no significant difference in the signal quality or in measurement performance related to the use of the textile–electronic connector in either implementation. The textile measuring system with a sensorized garment and connector was able to acquire ECG recordings with similar quality as those acquired using directly connected Ag/AgCl electrodes. The observed baseline wandering and powerline interference introduced by the system could be eliminated by proper filtering done when acquiring the ECG recordings.

The observed significant differences in volume resistivity are actually negligible when comparing these values with the volume resistivity value of copper. Conventional flex printed circuit connectors are made using polyimide substrates and printed copper traces with volume resistivity values as low as 0.67 mΩ/sq/mil [37]. The increased resistance observed for the MWCNT-based connector should not affect the measuring setup since it is <105 times smaller than the input impedance of the measurement instrumentation used for the electrical characterization [38]. Such a connector seems completely suitable for biopotential measurement applications but might not be a proper solution for other kinds of measurement modalities. In some cases, not only is voltage sensed but also electrical stimulation is required, e.g., electrical bioimpedance measurements, where low resistance in series with the measurement load is required to facilitate the current flow through the load to minimize measurement artifacts caused by stray capacitances [39] or electrode mismatch [40].

From the interfacing perspective with instrumentation amplifiers and other sensing electronic instrumentation, the resistance increase observed after folding is relatively too small to have any impact on the overall sensing function. In any case, a more adequate characterization study should be performed. In this regard, the authors believe that a folding test is actually very aggressive considering the intended purpose of the connector. Bending is probably a more realistic and adequate test for a connector meant to be attached to a recording device.

### 4.3. Functionalization of Textiles as Textile–Electronic Interconnections

CNT-based products have been used for functionalizing textiles for a decade [41], and screen printing processes have been shown to be effective for producing conductive textiles [42] with various purposes such as transmission lines [43] or radiofrequency antennas [44]. Combining CNTs with screen printing has been a successful approach to prototyping electronics on rubber substrates producing elastic diodes [45,46].

In the presented approach, the elasticity was preserved by PU, which is used in a twofold role as a binder and coating agent. First, as an elastic binding agent, polyurethane preserves the ability to stretch to the conductive paste; second, as a coating agent, it treats the fabric surface for printing. Coating the surface of a fabric with PU is one of the most common finishing techniques in textile manufacturing. In this case, the PU coating is critical for obtaining a successful print since the coating not only smooths the otherwise coarse surface of the fabric, which is essential for avoiding open-circuit connections, but also facilitates the bonding with the elastic MWCNT paste during the thermal curing.

The results suggest that the presented solution, combining screen printing, a PU–MWCNT blended conductive paste, and stretchable PU-coated commercially available fabrics, is feasible for prototyping textile–electronic interconnectors. Given the widespread and established use of the material and the processes in textile production, the presented approach has the required potential for enabling true series textile manufacturing.

The intended use of this combination of conductive and stretchable fabrics is to produce an elastic interconnection between a measuring device, typically wearable, with textile conductive pads in sensorized garments. Sensing or actuating electronic devices usually have a relatively small number of inputs/outputs (Bodykom 5, Hexoskin 9, Equivital EQ02 11), and an interconnector printed on fabric would suit these wearable devices nicely at Printed Circuit Board (PCB) -level interconnection. If attempting to interconnect other levels of integrations, like an Integrated Circuit (IC) pinout level, this type of technology will face size limitations that will prohibit its use. If the application requires a large number of interconnections, e.g., 16, given the current resolutions achievable with screen printing, it is reasonable to think that the proposed solution will suit such cases similarly to the ball grid array presented in [47].

Other authors have presented alternatives for textile electronic interconnections, but while being successful in decreasing the size of the interconnections, such a feature is achieved at the expense of elasticity [48].

The stretchability is provided by the conductive elastomer, PU, used in this formulation. Conductive elastomeric materials in the form of inks and pastes are the most common approaches as indicated in [49] 

### 4.4. Textile-Friendly Methods

The method of choice to transfer both conductive and elastic properties into the conformable planar surfaces of textile fabrics is printing technologies [50], especially when the alternative is micro-fabrication processes.

Not only the production cost should be taken into account when manufacturing sensorized garments—we also need to consider the adoption of the applied manufacturing techniques. Methods like soldering which are industrially mature but foreign to the textile manufacturing world, like in [48], will be more difficult to adopt in textile production lines than common methods already widely spread within textile manufacturing, like additive methods [50].

Much effort has been put into obtaining good textile electrodes with good sensing capabilities but very little effort has been devoted to developing methods and materials to support textile-friendly and easy-to-adopt techniques for textile–electronic integration. The use of well-known textile materials together with broadly accepted methods for textile functionalization reduces the risk when proposing a novel application of them combined, such as in this electronic interconnector fully integrated on a textile substrate.

### 4.5. Impact in Sensing Performace

The fact of producing a better-expressed QRS is an encouraging result because it suggests that the connector does not negatively impact the recordings; however, the authors believe that the better-expressed QRS is a product of using textile electrodes and not related to the use of the printed connector. The recordings suggest that the use of textile electrodes, with bigger areas than normal Ag/AgCl electrodes, produces a skin–electrode interface with lower resistance, increasing the cut-off frequency of the low-pass filter at the skin–electrode interface. Therefore, higher-frequency components are preserved, which in some cases can be seen as advantage for QRS segmentation but in other scenarios might produce noisier recordings. In such a case, there are plenty of digital noise canceling methods to choose from. 

## 5. Conclusions and Future Work

This study has presented positive results, especially from the functional perspective, since the sensing function was actually allowed. Now that that function has been shown to be possible, the next step is to investigate other material aspects further.

Future work will be focused on investigating the stability under certain degrees of deformation; assessing the need for encapsulation, if any; and evaluating the performance in other kinds of biosignal measurements or other applications such as digital interfacing, i.e., discrete voltage signals. In this study, the experimental protocol was selected to target the influence of the interconnection; therefore, sitting at rest was selected to avoid other sources of errors like motion artifacts. Now that interconnection shows promising results, assessment during exercise, as done in [51], should be done.

## Figures and Tables

**Table 1:** Mean resistance difference per number of folds.

Type of Connector	No. of Folds		
	10	20	50
MWCNT	−0.3	0.7	4.4
Silver	2.6	6.8	10.8

Difference reported in % for 10, 20, and 50 folds performed over a cylindrical rod of 2 mm radius.

**Table 2:** Characteristics of ECG recordings.

(Mean ± SD)	Gel Electrode and Cable Connection	Textile Electrodes with Printed Connectors	
		MWCNT Paste	Silver Paste
Powerline Interference RMS (mV)	0.02 ± 0.00	0.07 ± 0.01	0.05 ± 0.01
Short Time Baseline Wander (mV)	0.07 ± 0.02	1.60 ± 0.96	2.20 ± 1.40
R-peak Amplitude (mV)	0.70 ± 0.05	1.00 ± 0.06	0.99 ± 0.05
Correlation with Gel Electrode	N/A	0.96 ± 0.01	0.97 ± 0.00

## Appendix A. Connector Resistance after Folding

Table A1 contains the measurements obtained during the folding experiments performed on both connector types.

**Table A1:** Resistance difference after several bending cycles.

MWCNT Connector					Silver Connector				
*No. of Folds*	*0*	*10*	*20*	*50*	*No. of Folds*	*0*	*10*	*20*	*50*
***Connector #1***					***Connector #1***				
Line 1	11.5	11.2	11.5	12.0	Line 1	7.5	7.5	7.8	8
Line 2	11.1	10.9	11.4	11.7	Line 2	7.6	7.7	8	8.2
Line 3	9.2	9.4	9.1	9.8	Line 3	7.2	7.1	7.5	7.9
Line 4	9.8	10.0	9.9	10.2	Line 4	7.3	7.4	7.5	7.8
***Mean #1***	10.4	10.4	10.5	10.9	***Mean #1***	7.4	7.4	7.7	8.0
***Connector #2***					***Connector #2***				
Line 1	10.4	10.7	10.6	11.0	Line 1	8.1	8.3	8.5	8.8
Line 2	10.7	10.3	11.1	11.1	Line 2	7.8	8	8.1	8.5
Line 3	9.0	9.2	9.1	9.6	Line 3	7.3	7.5	7.9	8.1
Line 4	10.0	9.8	10.1	10.4	Line 4	7.3	7.3	7.6	8
***Mean #2***	10.0	10.0	10.2	10.5	***Mean #2***	7.6	7.8	8.0	8.4
***Connector #3***					***Connector #3***				
Line 1	11.0	10.7	10.6	11.4	Line 1	7.4	7.7	8	8.2
Line 2	11.2	11.0	11.5	11.7	Line 2	7.3	7.8	8	8.3
Line 3	9.6	9.8	9.7	10.0	Line 3	7.1	7.4	8.2	8.5
Line 4	10.3	10.4	10.1	10.4	Line 4	7.2	7.7	8.1	8.4
***Mean #3***	10.5	10.5	10.5	10.9	***Mean #3***	7.3	7.7	8.1	8.4
**Total mean**	**10.3**	**10.3**	**10.4**	**10.8**	**Total mean**	**7.4**	**7.6**	**7.9**	**8.2**
*Total Δ (%)*	*0*	*−0.3*	*0.7*	*4.4*	*Total Δ (%)*	*0*	*2.6*	*6.8*	*10.8*

## References

[1] Li L., Au W.M., Li Y., Wan K.M., Wan S.H., Wong K.S. (2010). Design of intelligent garment with transcutaneous electrical nerve stimulation function based on the intarsia knitting technique. Text. Res. J..

[2] Seoane F., Ferreira J., Alvarez L., Buendia R., Ayllon D., Llerena C., Gil-Pita R. (2013). Sensorized Garments and Textrode-Enabled Measurement Instrumentation for Ambulatory Assessment of the Autonomic Nervous System Response in the ATREC Project. Sensors.

[3] Yoo J., Yan L., Lee S., Kim H., Yoo H.-J. (2009). A wearable ECG acquisition system with compact planar-fashionable circuit board-based shirt. IEEE Trans. Inf. Technol. Biomed..

[4] Abtahi F., Ji G., Lu K., Rodby K., Seoane F. A knitted garment using intarsia technique for Heart Rate Variability biofeedback: Evaluation of initial prototype. Proceedings of the 2015 37th Annual International Conference of the IEEE Engineering in Medicine and Biology Society (EMBC).

[5] Nick Langston J., Connectivity T., Menlo Park C. Connectivity Challenges in Smart Textiles. https://www.telecomengine.com/article/connectivity-challenges-smart-textiles/.

[6] Miles C., Leslie W. (2010). Textile Printing.

[7] Perelaer J., Smith P.J., Mager D., Soltman D., Volkman S.K., Subramanian V., Korvink J.G., Schubert U.S. (2010). Printed electronics: The challenges involved in printing devices, interconnects, and contacts based on inorganic materials. J. Mater. Chem..

[8] Cohen S.R. (1974). A review of the health hazards from copper exposure. J. Occup. Environ. Med..

[9] Norman R.H. (1957). Conductive Rubbers and Plastics: Their Production, Application and Test Methods.

[10] Lötters J., Olthuis W., Veltink P., Bergveld P. (1997). The mechanical properties of the rubber elastic polymer polydimethylsiloxane for sensor applications. J. Micromech. Microeng..

[11] Li F., Qi L., Yang J., Xu M., Luo X., Ma D. (2000). Polyurethane/conducting carbon black composites: Structure, electric conductivity, strain recovery behavior, and their relationships. J. Appl. Polym. Sci..

[12] Gubbels F., Blacher S., Vanlathem E., Jérôme R., Deltour R., Brouers F., Teyssie P. (1995). Design of electrical composites: Determining the role of the morphology on the electrical properties of carbon black filled polymer blends. Macromolecules.

[13] Shin M.K., Oh J., Lima M., Kozlov M.E., Kim S.J., Baughman R.H. (2010). Elastomeric conductive composites based on carbon nanotube forests. Adv. Mater..

[14] Marinho B., Ghislandi M., Tkalya E., Koning C.E., de With G. (2012). Electrical conductivity of compacts of graphene, multi-wall carbon nanotubes, carbon black, and graphite powder. Powder Technol..

[15] Lin C.-T., Chang K.-C., Lin C.-L., Chiang C.-C., Lu S.-W., Chang S.-S., Lin B.-S., Liang H.-Y., Chen R.-J., Lee Y.-T. (2010). An intelligent telecardiology system using a wearable and wireless ECG to detect atrial fibrillation. IEEE Trans. Inf. Technol. Biomed..

[16] Nemati S., Ghassemi M.M., Ambai V., Isakadze N., Levantsevych O., Shah A., Clifford G.D. Monitoring and detecting atrial fibrillation using wearable technology. Proceedings of the 2016 38th Annual International Conference of the IEEE Engineering in Medicine and Biology Society (EMBC).

[17] Yang L., Lu K., Diaz-Olivares J.A., Seoane F., Lindecrantz K., Forsman M., Abtahi F., Eklund J.A. (2018). Towards smart work clothing for automatic risk assessment of physical workload. IEEE Access.

[18] Lu K., Yang L., Seoane F., Abtahi F., Forsman M., Lindecrantz K. (2018). Fusion of Heart Rate, Respiration and Motion Measurements from a Wearable Sensor System to Enhance Energy Expenditure Estimation. Sensors.

[19] Lu K., Yang L., Abtahi F., Lindecrantz K., Rödby K., Seoane F. (2019). Wearable Cardiorespiratory Monitoring System for Unobtrusive Free-Living Energy Expenditure Tracking.

[20] Seoane F., Mohino-Herranz I., Ferreira J., Alvarez L., Buendia R., Ayllón D., Llerena C., Gil-Pita R. (2014). Wearable biomedical measurement systems for assessment of mental stress of combatants in real time. Sensors.

[21] Zhang D. Wavelet approach for ECG baseline wander correction and noise reduction. Proceedings of the 2005 IEEE Engineering in Medicine and Biology 27th Annual Conference.

[22] Donoho D.L., Johnstone I.M. (1995). Adapting to unknown smoothness via wavelet shrinkage. J. Am. Stat. Assoc..

[23] Tikkanen P. (1999). Nonlinear wavelet and wavelet packet denoising of electrocardiogram signal. Biol. Cybern..

[24] Pan J., Tompkins W.J. (1985). A real-time QRS detection algorithm. Biomed. Eng. IEEE Trans..

[25] Willems J. (1985). Recommendations for measurement standards in quantitative electrocardiography. Eur. Heart J..

[26] Abtahi F., Seoane F., Lindecrantz K., Löfgren N. Elimination of ECG Artefacts in Foetal EEG Using Ensemble Average Subtraction and Wavelet Denoising Methods: A Simulation. Proceedings of the XIII Mediterranean Conference on Medical and Biological Engineering and Computing 2013.

[27] Lawrence I., Lin K. (1989). A concordance correlation coefficient to evaluate reproducibility. Biometrics.

[28] Kim S., Leonhardt S., Zimmermann N., Kranen P., Kensche D., Muller E., Quix C. Influence of contact pressure and moisture on the signal quality of a newly developed textile ECG sensor shirt. Proceedings of the 2008 5th International Summer School and Symposium on Medical Devices and Biosensors.

[29] Feng C., Liu K., Wu J.S., Liu L., Cheng J.S., Zhang Y., Sun Y., Li Q., Fan S., Jiang K. (2010). Flexible, stretchable, transparent conducting films made from superaligned carbon nanotubes. Adv. Funct. Mater..

[30] Lipomi D.J., Tee B.C.K., Vosgueritchian M., Bao Z. (2011). Stretchable organic solar cells. Adv. Mater..

[31] Lee P., Lee J., Lee H., Yeo J., Hong S., Nam K.H., Lee D., Lee S.S., Ko S.H. (2012). Highly stretchable and highly conductive metal electrode by very long metal nanowire percolation network. Adv. Mater..

[32] Xu F., Zhu Y. (2012). Highly conductive and stretchable silver nanowire conductors. Adv. Mater..

[33] Suikkola J., Björninen T., Mosallaei M., Kankkunen T., Iso-Ketola P., Ukkonen L., Vanhala J., Mäntysalo M. (2016). Screen-printing fabrication and characterization of stretchable electronics. Sci. Rep..

[34] Du D., Yang X., Yang Y., Zhao Y., Wang Y. (2019). Silver Nanowire Ink for Flexible Circuit on Textiles. Micromachines.

[35] Maheshwari N., Abd-Ellah M., Goldthorpe I.A. (2019). Transfer printing of silver nanowire conductive ink for E-textile applications. Flex. Print. Electron..

[36] 5000 Silver Conductor-DuPont. https://www.google.com.hk/url?sa=t&rct=j&q=&esrc=s&source=web&cd=1&ved=2ahUKEwi0yMvj65XlAhVYFogKHdaOC0YQFjAAegQIAxAC&url=https%3A%2F%2Fwww.dupont.com%2Fcontent%2Fdam%2Fdupont%2Fproducts-and-services%2Felectronic-and-electrical-materials%2Fdocuments%2Fprodlib%2F5000.pdf&usg=AOvVaw1LHS6FbW7Y5qrYZMvMsu1s.

[37] Khandpur R.S. (2005). Printed Circuit Boards: Design, Fabrication, Assembly and Testing.

[38] Lee J.W., Kim D.M., Park I.Y., Park H.J., Cho J.H. (2003). Characteristics of human skin impedance including at biological active points. IEEE Trans. Fundam. Electron. Commun. Comput. Sci..

[39] Scharfetter H., Hartinger P., Hinghofer-Szalkay H., Hutten H. (1998). A model of artefacts produced by stray capacitance during whole body or segmental bioimpedance spectroscopy. Physiol. Meas..

[40] Buendía R., Bogonez-Franco P., Nescolarde L., Seoane F. (2012). Influence of electrode mismatch on Cole parameter estimation from Total Right Side Electrical Bioimpedance Spectroscopy measurements. Med. Eng. Phys..

[41] Liu Y., Wang X., Qi K., Xin J. (2008). Functionalization of cotton with carbon nanotubes. J. Mater. Chem..

[42] Kazani I., Hertleer C., De Mey G., Schwarz A., Guxho G., Van Langenhove L. (2012). Electrical conductive textiles obtained by screen printing. Fibres Text. East. Eur..

[43] Locher I., Tröster G. (2007). Screen-printed textile transmission lines. Text. Res. J..

[44] Scarpello M.L., Kazani I., Hertleer C., Rogier H., Ginste D.V. (2012). Stability and efficiency of screen-printed wearable and washable antennas. IEEE Antennas Wirel. Propag. Lett..

[45] Sekitani T., Nakajima H., Maeda H., Fukushima T., Aida T., Hata K., Someya T. (2009). Stretchable active-matrix organic light-emitting diode display using printable elastic conductors. Nat. Mater..

[46] Sekitani T., Noguchi Y., Hata K., Fukushima T., Aida T., Someya T. (2008). A rubberlike stretchable active matrix using elastic conductors. Science.

[47] Mehmann A., Varga M., G K. A ball-grid-array-like electronics-to-textile pocket connector for wearable electronics. Proceedings of the 2015 ACM International Symposium on Wearable Computers.

[48] Poupyrev I., Gong N.-W., Fukuhara S., Karagozler M.E., Schwesig C., Robinson K.E. Project Jacquard: Interactive digital textiles at scale. Proceedings of the 2016 CHI Conference on Human Factors in Computing Systems.

[49] Tan E., Jing Q., Smith M., Kar-Narayan S., Occhipinti L. (2017). Needs and enabling technologies for stretchable electronics commercialization. MRS Adv..

[50] Seifert T., Sowade E., Roscher F., Wiemer M., Gessner T., Baumann R.R. (2015). Additive manufacturing technologies compared: Morphology of deposits of silver ink using inkjet and aerosol jet printing. Ind. Eng. Chem. Res..

[51] Meziane N., Yang S., Shokoueinejad M., Webster J., Attari M., Eren H. (2015). Simultaneous comparison of 1 gel with 4 dry electrode types for electrocardiography. Physiol. Meas..

